# Collective cell migration during human mammary gland organoid morphogenesis

**DOI:** 10.1063/5.0089767

**Published:** 2022-12-14

**Authors:** Franz P. Hutterer, Benedikt Buchmann, Lisa K. Engelbrecht, Andreas R. Bausch

**Affiliations:** Lehrstuhl für Biophysik E27, Center for Protein Assemplies (CPA) and Center of Organoid Systems (COS), Technical University Munich (TUM), Garching, Germany

## Abstract

Organ morphogenesis is driven by cellular migration patterns, which become accessible for observation in organoid cultures. We demonstrate here that mammary gland organoids cultured from human primary cells, exhibit oscillatory and collective migration patterns during their development into highly branched structures, as well as persistent rotational motion within the developed alveoli. Using high-resolution live-cell imaging, we observed cellular movement over the course of several days and subsequently characterized the underlying migration pattern by means of optical flow algorithms. Confined by the surrounding collagen matrix, characteristic correlated back-and-forth movements emerge due to a mismatch between branch invasion and cell migration speeds throughout the branch invasion phase. In contrast, alveolar cells exhibit continuous movement in the same direction. By modulating cell–cell adhesions, we identified collective migration as a prerequisite for sustaining these migration patterns both during the branching elongation process and after alveolus maturation.

## INTRODUCTION

Complex multicellular morphogenetic processes, such as embryogenesis, organogenesis, and tumorigenesis, are enabled by collective cellular behavior^1–3^ and orchestrated by biochemical^4,5^ and mechanical^6,7^ cues. Throughout a wide variety of processes, such as wound healing, branching morphogenesis, and tumorigenesis, collective migration is a key factor in enabling the formation of functional structures.^8^ In this case, long-ranged cellular patterns can emerge if correlated cell behavior is promoted by coupling of neighboring cells via cell–cell adhesions.^9^ This coupling leads to an integration of the individual traction forces and, thus, to a long-ranged stress field within the epithelial layer giving rise to high-order phenomena within the tissue.^10^

In order to recreate collective cell migration *in vitro*, cells are commonly cultivated on spatially confining 2D biopolymer patterns^9,11–14^ or inside simplified artificial 3D topologies.^15,16^ Within such confinement, collective cell migration is steered by the geometrical boundary conditions. It has been shown that cells on strip-like patterns exhibit a directed outgrowth as long as the strip diameter is narrower than the specific correlation length of the cells.^9^ In comparison, on circular and ring-shaped patterns with dimensions below, the correlation length cells perform rotational migration as long as they are coupled by cell–cell adhesions.^17^ Bridging the gap toward more complex systems, studies on multicellular spheroids have revealed complex rotational motion induced by the curvature^18^ that resembles the coherent angular motion previously found for small numbers of breast epithelial cells,^19^ which has been linked to basement membrane assembly,^20^ while cells under quasi-3D tubular confinement are reported to invade into the tube as a result of a rotational but directed migration.^15^ In organoid systems, this transition of linear to rotational motion has been shown to result from the isotropification of the tension field by the migrating cells.^21^

Such collective migration persists until the cell density increases above a critical threshold or the cell–cell adhesions maturate, which ultimately leads to a jamming of the cells and an inhibition of cell migration.^6,22^ Within biological tissues in 3D migrating cells interact strongly with the surrounding extracellular matrix (ECM), thus leading to remodeling and invasion of the matrix.^3,23^ During branching morphogenesis of human mammary gland organoids, the mechanical interaction of the cells with their extracellular matrix ultimately leads to the formation of a mechanically stable collagen cage surrounding the extending organoids.^24,25^ Such cell–ECM interactions and matrix remodeling play a central role in organ development and rely on the detailed nature of the collective cell migration patterns and their spatial–temporal coordination.

Herein, we report on collective cell migration during the morphogenesis of primary human mammary gland organoids. We used long-term confocal live-cell imaging to observe the fluorescently labeled nuclei of individual cells over time. Analysis of the movement by means of automatic and manual single cell tracking as well as the calculation of optical flow fields revealed different dynamics in various stages of development. In contrast to persistently rotating alveoli, the internal flow field within the spatially confined and elongated branches exhibits a collective back-and-forth migration of the cells, while the tip cells in the front of the branches largely show uncoupled migration behavior. By inhibiting cell–cell adhesion, matrix degradation, and cell contractility, we demonstrate that the collective cell patterns arise as a result of the relation between tip invasion speed into the ECM, the individual cell migration velocities in the branch and the subsequent localized jamming events.

## RESULTS

### Cells are highly motile within human mammary gland organoids

**FIG. 1.**
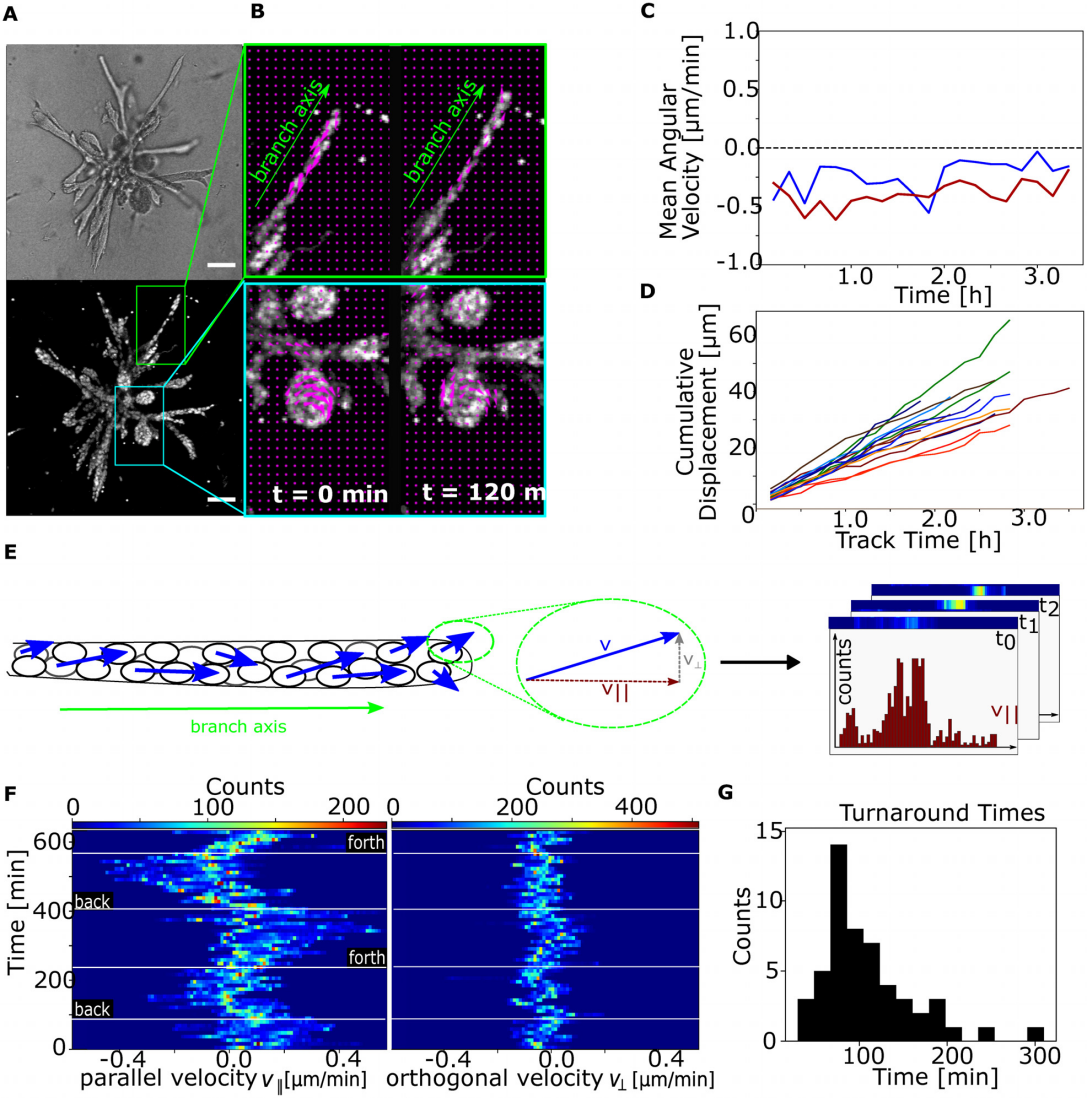
Experimental setup. (a) Bright-field and SiR-DNA stained nucleus signal of an exemplary mammary gland organoid (b) optical Flow reveals direction reversals in branches and rotations in alveoli. (c) Alveoli persistently rotating at nearly constant velocities without changing the rotation direction. (d) Single alveolar cells showing linear cumulative displacement, indicating persistent continuous migration at the single cell level. (e) By splitting the calculated velocities with respect to the branch axis and stacking the resulting histogram for all time steps the temporal change in the velocity distributions can be visualized. (f) The time-resolved histogram for a cylindrical branch reveals a distinct back-and-forth migration pattern parallel to the branch axis. In comparison, the orthogonal velocities were negligible. Scale bars, 100 *μ*m. (g) Histogram of the distribution of turnaround times for n = 52 turnaround events in n_b_ = 8 independent branches. The average turnaround time of the analyzed events is t = 108 min.

Human mammary gland organoids were prepared by seeding singularized human primary cells in floating collagen gels. Over the course of two weeks, single cells grew into highly branched and multicellular organoids with a diameter of up to 2 mm [Figs. 1(a) and Movie S1]. The growth of the organoids was finalized by the formation of spherical alveoli at the end of elongated branches, which arise due to an isotropification of the internal stress field within the branches.^21^ During branch elongation, their expansion is accompanied by a long-range and anisotropic deformation field in the surrounding collagen network, which is a result of the forces exerted by the organoids by means of internal cell migration. These plastic deformations ultimately lead to the formation of a mechanically stable collagen cage surrounding and stabilizing the organoids.^24^ This creates as spatial confinement for the cells within the organoid thus restricting and defining the cellular migration within the branches.

To analyze the resulting modes of cellular migration, we fluorescently labeled the cell nuclei using SiR-DNA stain and conducted live cell imaging of the organoids throughout all developmental stages. Due to heterogeneities in organoid morphology and age, the branch lengths ranged from 79 to 671 *μ*m, and the observed branch widths were between 15 and 63 *μ*m. The analyzed alveoli had diameters between 78 and 124 *μ*m and were all hollow in the center. The cells within organoids appeared to be highly motile throughout all developmental stages from day 7 until day 14 [Fig. S1(a)]. The average velocity across the analyzed cylindrical branches was around 0.14 *μ*m/min, reaching maximal values of up to 0.7 *μ*m/min. No statistically significant differences in average cell velocities were found between different organoid ages. Furthermore, the average and maximal velocities appeared to be independent of branch length and width [Fig. S1(b)]. We observed that the 3D cell migration within the branches was highly correlated with branch geometry. Cells within cylindrical branches moved mainly parallel to the branch axis and only exhibited a negligible migration orthogonal to the branch axis [Fig. 1(b)]. In comparison, the cells comprising the alveoli exhibited a rotational migration perpendicular to the main branch axis with speeds of up to 1 *μ*m/min [Figs. 1(b) and 1(c) and Movie S2]. The cumulative displacement of single cell tracks demonstrated a linear behavior, thus indicating continuous nonstop motion at the single cell level [Fig. 1(d)]. There was no prevailing chirality in rotation, with 45% of the analyzed alveoli (n = 20) rotating clockwise and 50% rotating counterclockwise. Only in 5% of the analyzed events a reversal of the rotation direction was observed. Therefore, once a rotational migration within the alveoli had been established, the cells persistently rotated in one direction. The observed migration modes in the elongated branches and spherical alveoli resembled those found on 2D-patterned surfaces having corresponding geometries.^9,11^ However, in contrast to these previous results in 2D systems, the cells in the branches did not exhibit a persistent migration in one direction but rather changed their migration direction over time. To reveal the temporal migration patterns, the optical flow fields obtained by analysis of the fluorescence live cell imaging data were projected and temporally resolved [Fig. 1(e)]: The resulting histograms of the parallel velocities for each frame were color-coded and displayed as a single line. Subsequent stacking of the individual lines illustrates the temporal development of the parallel velocity distribution within a branch. It, thereby, became apparent that the cells were not continuously migrating in only one direction, but rather exhibiting several phases, during which most cells within a branch collectively migrated either inwards or outward [Fig. 1(f)]. These back-and-forth migration patterns took place over several hours and were interrupted by comparatively short phases without motion and the subsequent migration reversals. The 52 analyzed turnaround events showed an average time between direction reversals of 108 min [Fig. 1(g)].

### Cells migrate collectively in space and time

**FIG. 2.**
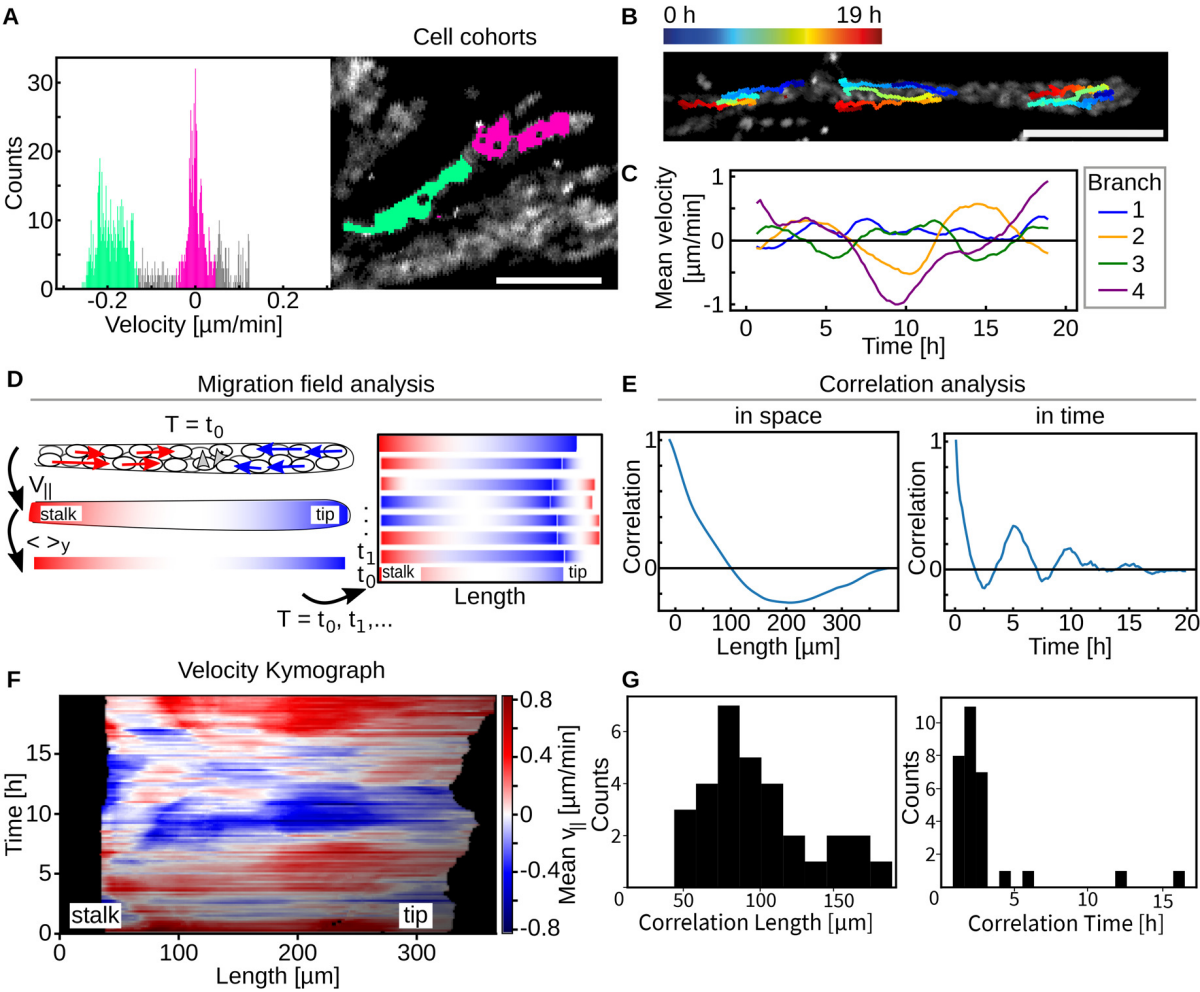
Spatio-temporal correlation analysis. (a) Peaks in the velocity distribution (green, magenta) correspond to spatially connected areas within the branch. (b) Individual single cell tracks show that single cells commuted within single branches and did not migrate into neighboring branches over time. (c) Branches of an organoid exhibit an oscillatory mean velocity. Their frequency and velocity are not correlated but rather independent off another. (d) For migration analysis in branches, the parallel component was averaged perpendicular to the branch axis. The resulting velocity line profiles were stacked for all timepoints revealing the spatiotemporal development of the oscillations. (e) Representative curves for the correlation analysis in space and time. The first zero crossing of correlation functions computed on mean-centered velocity kymographs was used to determine the correlation lengths and times. (f) Representative velocity kymograph of an extending branch. In red, outward directed phases appeared repeatedly after those of inwards oriented migration colored in blue. Large sections of the branch move in unison. (g) Distribution of correlation lengths and times in n_l_ = 31 and n_b_ = 28 branches. All scale bars 100 *μ*m.

A spatiotemporal analysis of the velocity fields was performed in order to further investigate the collective properties of the observed migration patterns. Given velocity histograms for a given branch exhibiting multiple distinct peaks at single time points, coloring pixels contributing to the individual peaks revealed clusters of collectively migrating cells [Fig. 2(a)]. By manually tracking single cells over multiple back-and-forth phases, it was confirmed that cells were confined to individual branches and thus, the organoid branches did not act as a large, interconnected flow system [Fig. 2(b)]. Instead, the migration patterns appeared to emerge locally and independently of other branches, leading to a non-synchronized movement of branches belonging to the same organoid [Fig. 2(c) and Movie S3].

In order to visualize the spatiotemporal development of the velocity field within a branch, parallel cell velocities were color-coded and subsequently averaged along the orthogonal branch axis, thus taking advantage of the cylindrical symmetry of the branches and the neglectable orthogonal components. Combining multiple of these one-dimensional representations of the branch velocity profile for different timesteps yielded velocity kymographs [Fig. 2(d)]. During phases of stable inwards or outward movement, large sections of the branches were moving in unison, which confirmed the temporal stability of the previously observed clusters, whereas uncorrelated movement was observed in the comparatively short turnaround phases [Figs. 2(f) and S2]. Temporal and spatial autocorrelation functions of the mean-centered kymographs were calculated to further examine the collectiveness of the underlying cell migration [Fig. 2(e)]. Examining the first zero-crossings of the resulting curves revealed average correlation times of 2 (±1) h and correlation lengths of between 65 and 105 *μ*m [Fig. 2(g)].

**FIG. 3.**
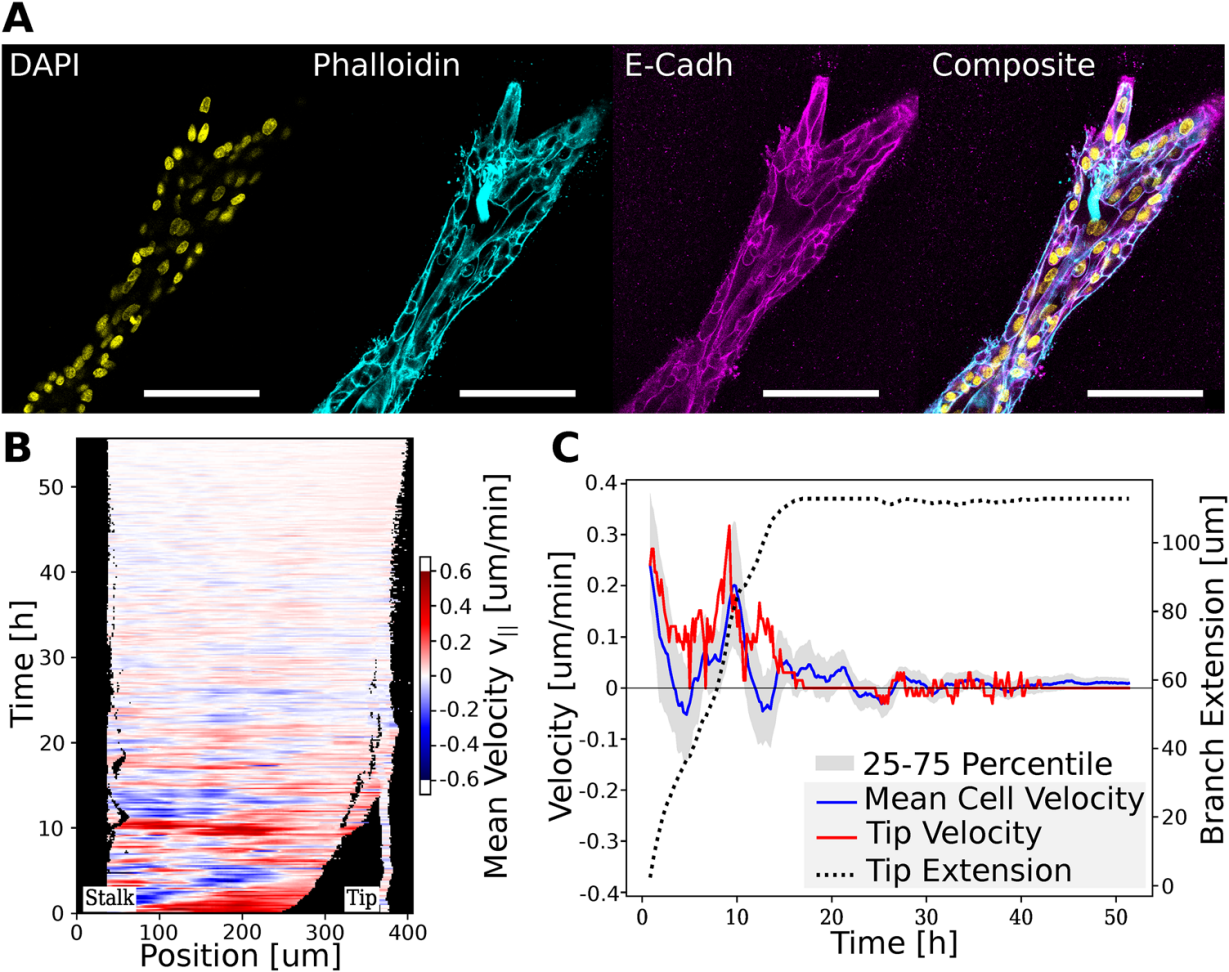
Cell–cell adhesion and contractility. (a) Close-up of an organoid branch stained for cell nuclei (DAPI), F-Actin (Phalloidin), and E-Cadherin (HECD1). Actin bundles that span several cells and expression of E-Cadherin across all cell membranes indicate high cellular coupling. (b) Velocity kymograph after E-Cadherin inhibition with HECD1. Initially, cells still showed back-and-forth like behavior, which comes to a standstill after approximately 15 h. (c) Mean cell velocity, tip velocity and tip extension over time after HECD1 treatment. As the mean cell velocities decreased, there was no further tip extension in the branches. Notably, the 25–75 percentiles also converged to zero indicating that virtually all cells in a branch stop migration. All scale bars, 100 *μ*m.

Given that antibody staining of f-actin indicated strong coupling between the cells in a branch, antibody staining of E-Cadherin (which is a transmembrane protein known for mediating cell–cell junctions) was performed to further understand the role of collective cell migration in branching morphogenesis [Fig. 3(a)]. The branch cells expressed high levels of localized E-Cadherin, which enables cellular crosstalk between neighboring cells and has been reported to be crucial to collective migration. We hypothesized that, in the densely packed branches, collective migration plays a key role in sustaining any cell movement. To test this hypothesis, the E-Cadherin antibody HECD1 was added to developed organoids, which were subsequently imaged for more than 50 h (Movie S4). Indeed, the inhibition of E-Cadherin led to a slowdown of the cells and prevented any collective cell migration within 15 and 24 h after treatment, allowing cells to only slowly fluctuate around their initial position [Figs. 3(b) and 3(c)].

### Mismatch between cell velocity and tip invasion induces back-and-forth migration

**FIG. 4.**
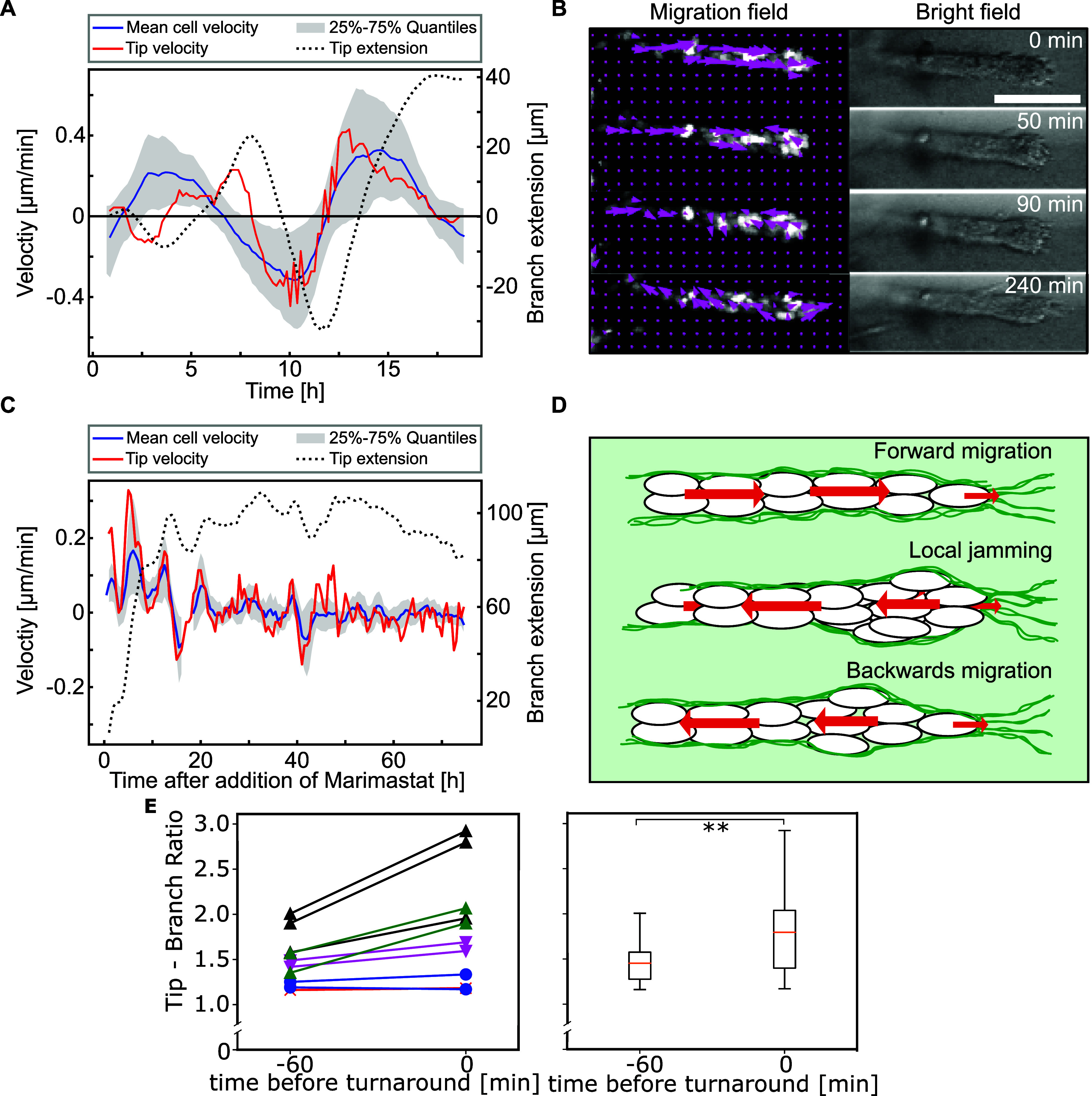
Mismatch between cell invasion and cell migration. (a) Mean cell velocity, tip velocity and relative tip extension over time. The tip velocity was predominantly within the 25–75 percentiles of the cell velocities. (b) Cell nuclei with optical flow field and bright-field images of an organoid branch. Cells jammed at the front and subsequently reversed their direction (c) metalloproteinase inhibition upon treatment with Marimastat led to a stagnation in matrix invasion and, ultimately, highly reduced cell velocities. (d) Schematic representation of the proposed mechanism for the emergence of back-and-forth-oscillations. Scale bar, 100 *μ*m (b). (e) Comparison of tip/branch thickness ratio 1 h before and at the point of direction reversal for ten turnaround events in five branches (colors) of four different organoids (markers). The tip thickness increased relative to the branch statistically significant with a p-value of 0.006 91.

To further understand these findings, the structural properties of the surrounding ECM must be considered. Notably, the organoids' branches were surrounded by a cage-like collagen structure as previously reported.^24^ This mechanically stable encasing, which had pores much smaller than average cell sizes created a barrier for the cells. As a result, cell migration within human mammary gland organoids should be understood as a form of cell migration under geometrical confinement. However, the spatial confinement of the branches in this system is not fixed. Instead, it is constantly being changed by the dynamic invasion process into the ECM. This process is facilitated by the tip cells, whose dynamics have been shown to be decoupled from the stalk cell migration. To investigate the effect of dynamic spatial confinement on cell migration patterns, we quantified the branch tip velocity and relative branch extension during organoid growth [Fig. 4(a)]. When comparing the determined invasion velocities of the branch tip with the migration velocity of the cells in the branch stalk, a clear mismatch became apparent. Throughout every inwards or outward directed migration phase, there were a substantial number of cells within a branch, which exceeded the tip invasion velocity. As a result, local jamming events were observed at the branch tips when the migrating stalk cells collided with the slower tip cells [Fig. 4(b) and Movie S5]. This region of high density brought cell migration to a halt and led to a direct reversal of the stalk cells, thus promoting the establishment of a new inward-directed migration phase during which the tip cells continue to pull on the surrounding collagen matrix. Importantly, by analyzing the tip/branch thickness ratio a statistically significant increase in relative tip thickness became apparent, further supporting the claim of increased cell crowding in the tip region at the time of migration reversal [Fig. 4(e)].

However, the ECM invasion process does not solely rely on mechanical remodeling; it also requires enzymatic matrix digestion.^26^ Cells secrete matrix metalloproteinases (MMPs) that degrade the surrounding collagen fibers, thus allowing the branch to increase in length. In order to demonstrate the impact of the observed velocity mismatch between invasion and migration velocities on branch contractions, drug treatments with Marimastat were performed on the organoids so as to inhibit matrix metalloproteinases. Cells were subsequently imaged for more than 70 h immediately after the drug was added in order to investigate the effect of decreased invasion speed on internal cell migration (see the supplementary material Movie S6).

During the initial hours of treatment, the cells still exhibited the characteristic back-and-forth patterns observed in the control. However, even though the cells continued to proliferate, the branch no longer significantly increased in length and cell velocities simultaneously decreased after 15–20 h [Fig. 4(c)]. Subsequently, cell migration came to a halt and, after 50 h of treatment, the branch tip slowly started to retract, indicating decreasing tension within the organoid branch.

## DISCUSSION

It has been previously reported that cells on 2D-patterned substrates exhibit migration patterns that are dictated by the geometrical boundary conditions. While cells seeded on strip-like patterns migrate mainly in parallel with respect to the main axis, circular patterns lead to the emergence of rotational migration.^11^ We found that similar migration modes can be observed during the growth of human mammary gland organoids in a three-dimensional matrix, in which the geometrical confinement is created by the dynamic formation of a self-built collagen cage around the organoids. As the branches span at most 5–7 cells in diameter during the branching morphogenesis phase in early organoid development and show a high degree of rotational symmetry with neglectable orthogonal velocities, we were able to quantify the most important aspects of the large-scale cell migration by analyzing the 2D projections of confocal image stacks. Here, the correlation length of cells migrating in extending branches was comparable to those found in 2D. In contrast to the previously described 2D and 3D experiments,^11,15^ the spatial confinement of extending organoid branches constantly changes over time and is remodeled by the tip cells themselves.^24^ Nevertheless, the matrix remodeling process at the invasion site imposes a limit on the maximum possible invasion speed of the branch tip. As a result, there is a significant mismatch between stalk migration velocities in the proximal part of the branch and the tip extension. Consequently, fast outward migrating cells will periodically jam at the branch tip, thus slowing down and ultimately changing their migration direction. Over time, this effect gives rise to an oscillatory back-and-forth migration mode, which is at the heart of branching morphogenesis in basal human mammary gland organoids. The dynamic coupling of the motion and force exertion of the branches to the ECM results in the mechano-plastic remodeling of the ECM—and leads to branch extension. This bidirectional interplay is not possible in Matrigel, due to the lack of mechanical plasticity, which might also explain different branching modes observed. While in pure Matrigel murine, mammary epithelial fragments of mouse cell lines have shown to form simple cyst-liked protrusions without a clear mechanical interaction of the cells with the matrix,^27,28^ already enriching the Matrigel with 30% prepolymerized collagen leads to branched structures of higher complexity.^29^ The mechanical plasticity of these networks is unknown and it remains to be determined what kind of cellular migration dynamics drive the branching morphogenesis in this model systems. To this end, the observed cellular migration patterns in the morphogenesis of human mammary gland organoids presented here may, thus, serve as a benchmark to further understand the morphogenesis of other mammary gland organoid systems as well as those of other organs with possibly even more complicated geometries.

## MATERIALS AND METHODS

### Organoid cultivation

Primary human mammary gland organoids were prepared following a previously published protocol (Linnemann). Freshly isolated human mammary gland epithelial cells from healthy women undergoing reduction mammoplasty were embedded in freely floating collagen I gels (Cornings). Branched organoids were formed by EpCAM+/CD49f+/CD10+ basal cells. The collagen concentration was set to 1.3 mg/ml. For the first five days, cells were cultivated in mammary epithelial growth medium (promocell MECGM) mixed with 3 *μ*M Y-27632 (Biomol), 10 *μ*M Forskolin (Biomol) and 0.5% FBS. After five days, the MECGM was enriched with 10 *μ*M Forskolin only. Organoids were grown at 37 °C at an atmosphere of 5% CO_2_ and 3% O_2_. Finally, organoids were imaged between days 6 and 12 to analyze the branching morphogenesis phase, while older organoids with fully formed alveoli were used to describe the rotational motion in the alveoli.

### Confocal live-cell imaging

Long-term live-cell imaging was conducted with a Leica SP8 lightning confocal microscope. Data were collected with Leica Application Suite X v. 3.57. The CO_2_ level (5%) and both temperature (37 °C) and humidity (80%) were controlled using a gas incubation system (Ibidi). In order to visualize the nuclei, cells were incubated with 10 *μ*M sirDNA (Spirochrome AG) 3 h prior to the measurements. Labeled nuclei were excited at a wavelength of 633 nm. The resulting signal was collected at an emission maximum of around 674 nm through a HCX PL APO 10×/0.40 CS dry objective. Dependent on the experiment the observation time varied between 12 and 72 h with time steps between each frame ranging from 5 to 30 min.

### Inhibitor treatments

Inhibitors were added at various developmental stages to the cell culture media. In particular, the media was enriched with the inhibitors directly prior to the experiment, which was then present throughout the whole measurement. Matrix degradation was inhibited by 10 *μ*M Marimastat (Merck), and cell–cell adhesion was lowered by blocking E-Cadherin via HECD1 (Abcam) in a dilution of 1:25.

### Immunofluorescence

Collagen gels were first washed with PBS solution, subsequently fixed with 4% paraformaldehyde for 15 min and finally rinsed twice with PBS solution again. The cell membranes were permeabilized with 0.2% Triton X-100 and blocked with 10% goat or donkey serum in 0.1% BSA. Regarding immunofluorescence stainings, the samples were incubated with primary and secondary antibodies diluted in 0.1% BSA. DAPI was used to visualize the cell nuclei, HECD1 (Abcam) was used to label E-cadherin and Phalloidin conjugated with Atto 647 (Sigma) was used to visualize the actin network. Alexa 488 (Life Technologies) was used as the secondary antibody for the E-Cadherin.

### Optical flow

Optical flow analysis was performed using the Gunnar–Farneback optical flow implementation in the Python 3 OpenCV library. Initial image processing and data analysis were performed using Python's scikit-image, SciPy, and OpenCV as well as ImageJ. The pixel displacements in between frames were determined by applying the Gunnar–Farneback optical flow implementation of the Python 3 OpenCV library to 2D projections of confocal microscopy stacks, thus enabling the calculation of a velocity vector for every pixel. First, noise reduction was performed on the images using a median filter. Second, regions of interest (ROIs) were defined together with a branch axis for every region. Third, the image sequence was analyzed using the optical flow algorithm and the resulting velocity vectors were split into parallel and orthogonal velocity components with respect to the defined branch axis.

### Single cell tracking

Single cell tracking was performed manually for 2D projected branches using FIJI. 3D-tracking of nuclei in alveoli was achieved using Leica AIVIA Version 10.0.9/2021.

### Calculation of correlation length and time

Correlation functions of the mean centered kymographs were computed using the numpy correlate function. For temporal correlation, the 1D correlation was calculated for every position along the branch axis and all obtained curves for a given ROI were subsequently averaged. Similarly, for the spatial correlation, single curves were computed for every timepoint and then averaged across all positions.

### Statistics and reproducibility

For statistical testing, p-values were calculated using two-tailed wilcoxon ranksum analysis as implemented Python's SciPy library. All experiments were repeated for at least three independently prepared organoid batches. The exact numbers of observed phenomena are reported in the figure legends.

## SUPPLEMENTARY MATERIAL

See the supplementary material for additional data as referenced in the article as well as live microscopy data and analysis movies.

## Data Availability

All relevant data supporting the key findings of this study are available within the article and its supplementary material files or from the corresponding author upon reasonable request.

## References

[1] P. Friedl and D. Gilmour , “ Collective cell migration in morphogenesis, regeneration and cancer,” Nat. Rev. Mol. Cell Biol. 10(7), 445–457 (2009). 10.1038/nrm2720 19546857

[2] E. Scarpa and R. Mayor , “ Collective cell migration in development,” J. Cell Biol. 212(2), 143–155 (2016). 10.1083/jcb.201508047 26783298 PMC4738384

[3] K. M. Yamada and M. Sixt , “ Mechanisms of 3D cell migration,” Nat. Rev. Mol. Cell Biol. 20(12), 738–752 (2019). 10.1038/s41580-019-0172-9 31582855

[4] L. Tahtamouni *et al.*, “ Molecular regulation of cancer cell migration, invasion, and metastasis,” Anal. Cell. Pathol. 2019, 1356508. 10.1155/2019/1356508 PMC6536998 31218208

[5] C. Y. Chung , S. Funamoto , and R. A. Firtel , “ Signaling pathways controlling cell polarity and chemotaxis,” Trends Biochem. Sci. 26(9), 557–566 (2001). 10.1016/S0968-0004(01)01934-X 11551793

[6] S. Menon and K. A. Beningo , “ Cancer cell invasion is enhanced by applied mechanical stimulation,” PLoS One 6(2), e17277 (2011). 10.1371/journal.pone.0017277 21359145 PMC3040771

[7] C. T. Mierke , “ Physical role of nuclear and cytoskeletal confinements in cell migration mode selection and switching,” AIMS Biophys. 4(4), 615–658 (2017). 10.3934/biophy.2017.4.615

[8] A. Brugues *et al.*, “ Forces driving epithelial wound healing,” Nat. Phys. 10(9), 683–690 (2014). 10.1038/nphys3040 27340423 PMC4915550

[9] S. R. Vedula *et al.*, “ Emerging modes of collective cell migration induced by geometrical constraints,” Proc. Natl. Acad. Sci. U.S.A. 109(32), 12974–12979 (2012). 10.1073/pnas.1119313109 22814373 PMC3420172

[10] X. Trepat *et al.*, “ Physical forces during collective cell migration,” Nat. Phys. 5(6), 426–430 (2009). 10.1038/nphys1269

[11] K. Doxzen *et al.*, “ Guidance of collective cell migration by substrate geometry,” Integr. Biol. 5(8), 1026–1035 (2013). 10.1039/c3ib40054a 23784144

[12] C. Pérez-González *et al.*, “ Mechanical compartmentalization of the intestinal organoid enables crypt folding and collective cell migration,” Nat. Cell Biol. 23(7), 745–757 (2021). 10.1038/s41556-021-00699-6 34155382 PMC7611697

[13] D. Mohammed *et al.*, “ Substrate area confinement is a key determinant of cell velocity in collective migration,” Nat. Phys. 15(8), 858–866 (2019). 10.1038/s41567-019-0543-3

[14] C. G. Rolli *et al.*, “ Switchable adhesive substrates: Revealing geometry dependence in collective cell behavior,” Biomaterials 33(8), 2409–2418 (2012). 10.1016/j.biomaterials.2011.12.012 22197568

[15] W. Xi *et al.*, “ Emergent patterns of collective cell migration under tubular confinement,” Nat. Commun. 8(1), 1517 (2017). 10.1038/s41467-017-01390-x 29142242 PMC5688140

[16] L. Alaimo *et al.*, “ Engineering slit-like channels for studying the growth of epithelial tissues in 3D-confined spaces,” Biotechnol. Bioeng. 117(9), 2887–2896 (2020). 10.1002/bit.27446 32484903

[17] F. J. Segerer *et al.*, “ Emergence and persistence of collective cell migration on small circular micropatterns,” Phys. Rev. Lett. 114(22), 228102 (2015). 10.1103/PhysRevLett.114.228102 26196648

[18] T. Brandstätter *et al.*, “ Curvature induces active velocity waves in rotating multicellular spheroids,” arXiv:2110.14614 (2021). 10.1038/s41467-023-37054-2 PMC10039078 36964141

[19] K. Tanner *et al.*, “ Coherent angular motion in the establishment of multicellular architecture of glandular tissues,” Proc. Natl. Acad. Sci. U.S.A. 109(6), 1973–1978 (2012). 10.1073/pnas.1119578109 22308439 PMC3277511

[20] H. Wang *et al.*, “ Rotational motion during three-dimensional morphogenesis of mammary epithelial acini relates to laminin matrix assembly,” Proc. Natl. Acad. Sci. 110(1), 163–168 (2013). 10.1073/pnas.1201141110 23248267 PMC3538193

[21] P. A. Fernández *et al.*, “ Surface-tension-induced budding drives alveologenesis in human mammary gland organoids,” Nat. Phys. 17(10), 1130–1136 (2021). 10.1038/s41567-021-01336-7 35721781 PMC7612858

[22] S. Garcia *et al.*, “ Physics of active jamming during collective cellular motion in a monolayer,” Proc. Natl. Acad. Sci. 112(50), 15314–15319 (2015). 10.1073/pnas.1510973112 26627719 PMC4687586

[23] Y. L. Han *et al.*, “ Cell contraction induces long-ranged stress stiffening in the extracellular matrix,” Proc. Natl. Acad. Sci. 115(16), 4075–4080 (2018). 10.1073/pnas.1722619115 29618614 PMC5910866

[24] B. Buchmann *et al.*, “ Mechanical plasticity of collagen directs branch elongation in human mammary gland organoids,” Nat. Commun. 12(1), 2759 (2021). 10.1038/s41467-021-22988-2 33980857 PMC8115695

[25] B. Buchmann , P. Fernández , and A. R. Bausch , “ The role of nonlinear mechanical properties of biomimetic hydrogels for organoid growth,” Biophys. Rev. 2(2), 021401 (2021). 10.1063/5.0044653 PMC7612859 35722505

[26] J. E. Fata , Z. Werb , and M. J. Bissell , “ Regulation of mammary gland branching morphogenesis by the extracellular matrix and its remodeling enzymes,” Breast Cancer Res. 6(1), 1–11 (2004). 10.1186/bcr634 14680479 PMC314442

[27] A. J. Ewald *et al.*, “ Collective epithelial migration and cell rearrangements drive mammary branching morphogenesis,” Dev. Cell 14(4), 570–581 (2008). 10.1016/j.devcel.2008.03.003 18410732 PMC2773823

[28] A. J. Ewald *et al.*, “ Mammary collective cell migration involves transient loss of epithelial features and individual cell migration within the epithelium,” J. Cell Sci. 125(11), 2638–2654 (2012). 10.1242/jcs.096875 22344263 PMC3403234

[29] M. Caruso *et al.*, “ A mammary organoid model to study branching morphogenesis,” Front. Physiol. 13, 826107 (2022). 10.3389/fphys.2022.826107 35399282 PMC8988230

